# A Three-Wave Longitudinal Study of Moderated Mediation Between High-Performance Work Systems and Employee Job Satisfaction: The Role of Relational Coordination and Peer Justice Climate

**DOI:** 10.3389/fpsyg.2020.00792

**Published:** 2020-05-15

**Authors:** Sajid Haider, Carmen De-Pablos-Heredero, Monica De-Pablos-Heredero

**Affiliations:** ^1^Department of Management Sciences, COMSATS University Islamabad, Vehari Campus, Islamabad, Pakistan; ^2^ESIC Business & Marketing School and Universidad Rey Juan Carlos, Madrid, Spain; ^3^ESIC Business & Marketing School, Madrid, Spain

**Keywords:** high-performance work systems, job satisfaction, relational coordination, peer justice climate, longitudinal, moderated mediation

## Abstract

Existing literature lacks studies that examine the indirect effect of high-performance work systems (HPWSs) on employee job satisfaction through employee–employee relations. Moreover, less is known about the boundary conditions of this indirect effect. This study sought to longitudinally examine the mediating role of a specific form of employee–employee relations—relational coordination—in the relationship between HPWS and job satisfaction. Data were collected in three waves from the employees of commercial banks (*N* = 322). Partial least squares structural equation modeling was used for data analysis. Results from multiple linear autoregressive longitudinal analysis indicate that HPWSs predict relational coordination, which in turn partially mediates the HPWS–job satisfaction relationship. Perceptions of peer justice climate provide boundary conditions for the aforementioned mediating effect. This study contributes to existing literature by explaining moderated-mediation mechanisms through which HPWSs predict employee job satisfaction. Managers can strengthen the effect of HPWS on employee–employee relations and subsequent effect on employee job satisfaction by promoting peer justice climate in organizations.

## Introduction

High-performance work systems (HPWSs) have long been recognized as a means of firm performance (Fu et al., 2019). High-performance work systems refer to “a system of HR [human resource] practices designed to enhance employees” skills, commitment, and productivity in such a way that employees become a source of sustainable competitive advantage” (Datta et al., 2005, p. 136). This system of HR practices is generally induced by organizations to implement strategic human resource management. One remarkable progress in understanding the effect of strategic human resource management in the last two decades has been the rise of studies relating HPWS with employee outcomes (Agarwal and Farndale, 2017; Cooke et al., 2019; Martinaityte et al., 2019). Within employee level phenomena, job satisfaction has received greater attention as a consequence of HPWSs (Brinck et al., 2019) because it enhances employee productivity and performance (Argyle, 1989; Lucas and Diener, 2003). Job satisfaction can be defined as “a pleasurable or positive emotional state resulting from the appraisal of one’s job or work experiences” (Locke, 1976, p. 1300). Based on motivational theories, it is presumed that HPWSs enhance employee well-being and satisfaction by providing them appropriate work conditions (Zhang et al., 2018; Brinck et al., 2019).

Given the importance of mediators as a “generative mechanism through which the focal independent variable is able to influence the dependent variable of interest” (Baron and Kenny, 1986, p. 1173), many good works appeared that examined the mechanisms through which HPWSs exert their effect on employee job satisfaction. For example, Liu et al. (2016) found partial mediation of organizational identification in this relationship. Perceived organizational support was found as a strong mediator between HPWS–job satisfaction relationship (García-Chas et al., 2016). Chen et al. (2016) reported psychological efficacy as a significant mediator of the relationship between HPWSs and job satisfaction. Similarly, procedural justice, interactional justice, and organizational role stress mediated the HPWS–job satisfaction relationship (Wu and Chaturvedi, 2009; Heffernan and Dundon, 2012; Garg, 2015).

However, these studies (and other similar work) remained mainly focused on examining the organizational- and individual-level phenomena and largely ignored employee–employee level episodes that may explain the relationship between HPWSs and job satisfaction. More specifically, employee–employee relations have been ignored as a mechanism between HPWSs and employee job satisfaction (Evans and Davis, 2005). Employees working under a same set of high-performance work practices are expected to have shared understanding of and attitude toward interpersonal exchange relationships in organization (Gong et al., 2010). This shared understanding strengthens organization’s internal social structure, nourishes employment relationship, and, consequently, enhances employee job satisfaction (Requena, 2003; Evans and Davis, 2005; Chen et al., 2018). Given that HPWSs affect employees’ interpersonal relationships in organizations, and these relationships increase employee job satisfaction, it can be stated that employee relations can mediate the relationship between HPWSs and employee job satisfaction (Evans and Davis, 2005; Haider et al., 2019a). Thus, employee–employee relationships constitute a mediating mechanism through which HPWSs affect organizational and employee outcomes (Gittell et al., 2010).

Existing literature has discussed employee–employee relationships in various forms such as social capital (Evans and Davis, 2005), coworker exchange (Sherony and Green, 2002), mindful interacting (Vogus, 2006), social networks (Collins and Clark, 2003), relational coordination (Gittell, 2000), and so on. Among the diverse forms of employee relations in organization, relational coordination provides a unique framework for understanding interpersonal relationships at work settings as it believes that employees’ relational and communication ties interact with each other to achieve coordination during a work process (Gittell et al., 2010). According to Gittell (2002), “Relational coordination is a mutually reinforcing process of interaction between communication and relationships carried out for the purpose of task integration” (p. 301). Relational coordination is receiving the researchers’ attention as it provides a unique perspective on high-quality employee–employee relations characterized by the interaction between employee communication and relational ties and focuses on microdynamics to develop collective identity among organizational members (Gittell, 2006; Carmeli and Gittell, 2009).

Relational coordination has been examined as a mediator of the relationship between HPWSs and quality and efficiency outcomes in healthcare settings (Gittell et al., 2010). In another study, employee job satisfaction was significantly predicted by relational coordination (Gittell et al., 2008). The results of these studies suggest that HPWSs predict relational coordination, and relational coordination predicts employee job satisfaction. Given that, a mediating effect of relational coordination can be expected between HPWSs and job satisfaction. To the researchers’ knowledge, no previous study has examined the indirect effect of HPWSs on employee job satisfaction through relational coordination.

Furthermore, contemporary literature suggests that the contingent nature of indirect effect (or boundary conditions) needs to be examined for understanding “when that effect exists and when it does not” (Hayes, 2018, p. 4). Insights from organizational justice literature propound that employees’ justice perceptions play a vital role in developing cooperation among coworkers (Li and Cropanzano, 2009; Li et al., 2013). Specifically, peer justice climate, “defined as team-level judgments of the fairness with which coworkers generally treat one another” (Li et al., 2013, p. 563), is positively associated with workplace cooperation and communication, which promote knowledge sharing, group coordination, and interpersonal relationships (Cropanzano et al., 2011; Li et al., 2013). This study proposes that our mediator, that is, relational coordination (characterized by communication and relationships), may be affected by the level of peer justice climate in organization. In other words, employees working in a peer justice climate are likely to develop positive interactions due to greater satisfaction with coworkers (Haider et al., 2018). Therefore, they are highly likely to exercise relational coordination among each other.

Findings of various empirical studies suggest that justice climate may interact with diverse organizational and individual phenomena to predict outcomes at employee and organizational level (Li et al., 2013). However, the moderating effect of peer justice climate on the relationship between HPWSs and relational coordination has not been found in existing literature. As peer justice climate affects communication and interpersonal relationships among coworkers (Li et al., 2013), this study posits that the effect of HPWSs on relational coordination may differ at different levels of peer justice climate in organization. Given that, it can be stated that the indirect effect of HPWSs on employee job satisfaction through relational coordination is moderated by peer justice climate.

Moreover, previous studies have examined the mediating mechanisms between HPWSs and job satisfaction in cross-sectional study designs, which are not well suited to test causal effects such as mediation (Cole and Maxwell, 2003; Maxwell and Cole, 2007). High-performance work systems exert their effect on employee outcomes over time (Birdi et al., 2008; Piening et al., 2013; Shin and Konrad, 2017). In other words, HPWSs affect employee job satisfaction by changing the nature of employee–employee relations over time. Given the mediational nature of this relationship, there is need to “collect data in a fashion that allows time to elapse between the theoretical cause and its anticipated effect” (Cole and Maxwell, 2003, p. 561). So, we believe that longitudinal (rather than cross-sectional) study designs should be used for determining true causal relationships in a mediation model of HPWSs and employee job satisfaction.

The main objective of this study was to examine the mediating role of relational coordination between HPWS–job satisfaction relationship in a three-wave longitudinal study design. In addition, this study sought to examine the role of peer justice climate as a boundary condition for the aforementioned mediation process. Specifically, this study examined a moderated mediation model where peer justice climate increases employee job satisfaction by strengthening the effect of HPWSs on relational coordination among coworkers.

## Theory and Hypotheses

**FIGURE 1:**
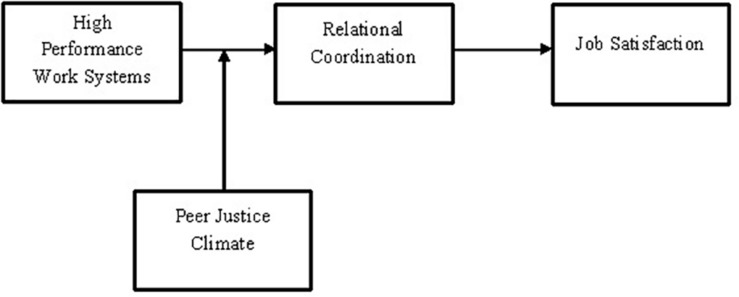
Theoretical model.

Figure 1 shows this study’s theoretical model where HPWSs predict job satisfaction through relational coordination. This indirect effect is moderated by peer justice climate. Specifically, peer justice climate moderates the effect of HPWSs on relational coordination. The variables and the nature of relationships depicted in this model are relevant to two major theoretical frameworks discussed in organizational studies: social exchange theory (SET) (Blau, 1964) and contingency theory of organizations (Drazin and Van de Ven, 1985). SET (Blau, 1964) has long been recognized as a framework for understanding social relationships in organizations. The generic model of social exchange suggests that any initiating action leads to a reciprocal response from the person/s for whom the action was initiated (Cropanzano et al., 2017). Based on this idea, the researchers in organizational psychology and human resource management have argued that the implementation of management practices positively affects social exchanges in an organization, which, subsequently, generate attitudinal and behavioral responses from employees (Takeuchi et al., 2007; Gong et al., 2010). These insights guide this study to suggest that HPWSs generate employees’ attitudinal response (i.e., job satisfaction) by positively effecting employee relations in organization (i.e., relational coordination). The moderator’s (peer justice climate) effect in Figure 1 is relevant to the contingency theory of organizations, which describes that the organization’s contextual factors may affect the intensity of relationships among diverse organizational phenomena (Drazin and Van de Ven, 1985). This study argues that peer justice climate, as an important contextual factor, may affect the indirect relationship between HPWSs and employee job satisfaction through relational coordination.

### HPWSs and Relational Coordination

While examining the processes underlying social exchange in work organization, Gittell (2002) described that relational coordination occurs through frequent, timely, accurate, and problem-solving communication, which is supported by shared knowledge, shared goals, and respect for each other. Gittell et al. (2008) and Gittell et al. (2010) argued that organizations’ use of HPWSs supports relational coordination among employees and subsequently affects organizational and employee outcomes such as quality, efficiency, and job satisfaction. This provides the idea that relational coordination resulting from the use of HPWSs may be vital for deciding if any positive employee outcomes appear from the social exchange processes at workplace. We connote that a reason why HPWSs predict job satisfaction is that the employees may perceive that organization’s use of HPWSs provides them an opportunity to develop workplace relationships. Consequently, this perception may provoke employee job satisfaction.

The relationship between relational coordination and employee job satisfaction has been supported in recent research (Gittell et al., 2008; Margalina et al., 2014). However, can relational coordination also explain why HPWSs predict job satisfaction? In order to support the assumption that relational coordination mediates the HPWS–job satisfaction relationship, we must support HPWSs as antecedents of relational coordination. Gittell et al. (2010) focused on six high-performance work practices: selection, conflict resolution, performance measurement, rewards, meetings, and boundary spanners. Gittell et al. (2010) explained that “boundary spanners are staff members whose primary task is to integrate the work of other people around a project, process, or customer” (p. 493). Gittell et al. (2010) developed theory-driven relationships between the aforementioned practices and relational coordination. They described that these practices “are expected to foster relational coordination, which is reflected in the frequency, timeliness, accuracy, and problem-solving nature of communication among employees and the degree to which their relationships are characterized by shared goals, shared knowledge, and mutual respect” (p. 494).

Consistent with Gittell et al. (2010), Riaz (2016) found a significant positive relationship between HPWSs and relational coordination. These thoughts are also consistent with Mossholder et al. (2011) and Vogus (2006), who described that high-performance HR practices promote employee relationships with their coworkers. Accordingly, it can be stated that employees could consider HPWSs as their organization’s relationship-enhancing activity and obtain greater job satisfaction in this relational environment. From a social capital perspective, Jiang and Liu (2015) described that HPWSs promote shared cognitive systems and “enhance the relationships among members within an organization and improve interpersonal communication and coordination” (p. 130). This idea is consistent with Evans and Davis (2005), who noted that “HPWSs positively influence the internal social structure by facilitating bridging network ties, generalized norms of reciprocity, shared mental models role, making, and organizational citizenship behavior” (p. 758). It shows that organization’s use of HPWSs, in fact, is the height of human resource management, which, according to Guest (1987), maximizes integration and collective relations in organizations. Based on above discussion, our first hypothesis is:

**Hypothesis 1:** High-performance work systems are positively associated with relational coordination.

### Mediating Role of Relational Coordination

Social exchange theory (Blau, 1964) comes up with a valuable framework for explaining the dynamic relationships in our research model as it concentrates on the continuous reciprocal exchange between organizational inputs (i.e., HR practices) and employees’ attitudinal and behavioral responses (Piening et al., 2013). The norm of reciprocity in SET suggests that organization’s use of high-performance work practices may develop, over time, employees’ shared understanding of exchange relationships, not only with their organization but also with their colleagues (Evans and Davis, 2005; Gittell et al., 2010; Gong et al., 2010; Chun et al., 2013). Based on Gittell et al.’s (2010) interpretation of the reciprocity emerging from organization’s use of high-performance work practices, it can be stated that HPWSs predict employee–employee relationships (i.e., relational coordination) over time. In other words, employees’ perceptions of HPWSs at one-point of time would lead relational coordination among coworkers at a future point of time. Subsequently, the dynamic nature of this relationship enhances employee job satisfaction over time.

In order to explain why HPWSs may affect employee job satisfaction, through relational coordination, we also used Gittell et al.’s (2010) model of HPWSs. Gittell et al.’s (2010) model includes following high-performance work practices: selection, conflict resolution, performance measurement, rewards, meetings, and use of boundary spanners. These authors proposed that employee selection, based on their ability to work in teams and person-organization fit, enhances mutual respect among coworkers. Organizations’ use of “selection” tends to enhance employee job satisfaction by stimulating high-quality exchange relationships, which are characterized by mutual respect (Sherony and Green, 2002; Janssen and Van Yperen, 2004). Given the importance of mutual respect in enhancing employee job satisfaction (Gittell et al., 2008; Clarke and Mahadi, 2017), we can expect that HPWSs facilitate employee job satisfaction at least partially through their effect on mutual respect among coworkers.

The existence of “conflict resolution” mechanisms in organizations is likely to encourage knowledge sharing and mutual respect among fellow workers (Gittell et al., 2010). Conflict resolution is likely to increase employee job satisfaction by promoting those cultures that invigorate mutual respect and knowledge sharing (Trivellas et al., 2015). Recognizing the fact that mutual respect and knowledge sharing predict employee job satisfaction (Gittell et al., 2008; Trivellas et al., 2015; Kianto et al., 2016), it can be assumed that HPWSs predict employee job satisfaction through their effect on mutual respect and knowledge sharing among fellow workers.

Organizations’ use of “performance measurement” practice is believed to “strengthen the shared goals and problem-solving communication dimensions of relational coordination” (Gittell et al., 2010, p. 493). Gittell et al. (2010) defined performance measurement as accountability for outcomes. Accountability is quite a suitable practice to augment employee job satisfaction because it encourages those behaviors (i.e., organizational citizenship behavior) that play a vital role in employees’ goal sharing and problem-solving (Lee et al., 1991; Turnipseed and Rassuli, 2005; Hall et al., 2009). Once we acknowledge that goal sharing and problem-solving communication enhance job satisfaction (Gittell et al., 2008; Hall et al., 2009), it is easy to accept that HPWSs increase employee job satisfaction by affecting goal-sharing and problem-solving communication.

“Meetings,” “rewards,” and “boundary spanners” enhance goal sharing and knowledge sharing and encourage, overall, the communication dimensions of relational coordination (Gittell et al., 2010). These practices may increase employee job satisfaction by strengthening their effect on relational coordination. Insights from Carmeli and Gittell (2009) provide the same argument as they presumed that “high-quality relationships as manifested in shared goals, shared knowledge, and mutual respect create a positive social context in which people feel safe to perform and act” (p. 714). This positive social context emerges when organizations use HPWSs, as a result of which the relational coordination is promoted (Gittell et al., 2010). Accordingly, it can be stated that a reason why HPWSs predict employee job satisfaction is that HPWSs may affect employee’s perceptions that they are working in a psychological safe environment characterized by high-quality relationships and communication (Carmeli and Gittell, 2009).

In order to ascertain whether relational coordination explains the relationship between HPWSs and employee job satisfaction, the following hypothesis was tested:

**Hypothesis 2:** Relational coordination mediates the relationship between HPWSs and employee job satisfaction.

### Moderating Role of Peer Justice Climate

Peer justice climate provides strong foundations for establishing good communication, coordination, and interpersonal relationships among coworkers (Li et al., 2013). Previous research suggests that perceptions of peer justice promote organizationally desired behaviors such as organizational citizenship behavior and team satisfaction and performance by improving cooperative teamwork processes (i.e., communication, cooperation, coordination, cohesion, etc.) among coworkers (Cropanzano et al., 2011; Li et al., 2013). Peer justice also enhances group learning behavior when it is influenced by group ethical conduct (Walumbwa et al., 2017). These insights from existing literature suggest that relational coordination as an indicator of teamwork quality (Heredero et al., 2015) is greatly influenced by peer justice climate in organization. Literature also suggests that peer justice climate has the ability to change employee and organizational outcomes when it interacts with the antecedents of these outcomes (Li et al., 2013). Given that both HPWSs and peer justice influence relational coordination, and justice climate moderates various relationships in organizations, it can be expected that peer justice climate may change the effect of HPWSs on relational coordination when it interacts with HPWS.

Contingency theory of organizations (Lawrence and Lorsch, 1967; Drazin and Van de Ven, 1985) suggests that “boundary conditions specify the ranges over which a relationship is expected to hold” (Drazin and Van de Ven, 1985, p. 514). Peer justice climate, as a unique organizational context, may “affect the occurrence, meaning, and outcomes of certain behaviors” (Heslin, 2009, p. 133) and consequently may provide boundary conditions for the relationship between HPWSs and relational coordination. In other words, the effect of HPWSs on relational coordination will be stronger when employees strongly exhibit the dimensions of relational coordination (i.e., frequent, timely, accurate, and problem-solving communication and shared knowledge, shared goals, and mutual respect) as a result of peer justice climate.

Similarly, the notion of reciprocity in SET suggests that employees “tend to reciprocate beneficial treatment they receive with positive work-related behaviors” (Hekman et al., 2009). According to Li et al. (2013), “when individuals are treated fairly by their teammates (PJC)…, and when these feeling are shared among team members, they may consider the work environment as pleasant and satisfactory.” As “social exchange tends to engender feelings of personal obligation, gratitude, and trust” (Blau, 1964, p.94), peer justice can make employees reciprocate in the form positive interpersonal relationships (mutual respect, knowledge sharing, and goal sharing) and fair communication (timely, accurate, frequent, and problem-solving communication). Given that peer justice climate enhances relational coordination, the effect of HPWSs on relational coordination will be stronger when employees’ perceptions of peer justice are high, and this relationship will be weak when these perceptions are low. So, it can be argued that the benefits of HPWSs in improving relational coordination among coworkers increase when they interact with peer justice climate.

Considering peer justice climate as a moderator of the relationship between HPWSs and relational coordination, this study argues that peer justice climate increases employee job satisfaction by strengthening the effect of HPWSs on relational coordination. In other words, peer justice climate makes a difference in specifying the effect of HPWSs on employee job satisfaction through relational coordination. We contend that a positive peer justice climate extends the satisfaction-enhancing benefits of HPWSs by strengthening relational coordination among coworkers. Although HPWSs enhance employee job satisfaction by influencing relational coordination among coworkers, the strength of this indirect effect depends on peer justice climate. This discussion leads us to formulate the following hypothesis:

**Hypothesis 3:** Peer justice climate moderates the indirect effect of HPWSs on employee job satisfaction through relational coordination.

## Materials and Methods

### Sample and Procedures

Participants were employees from commercial banks in Southern Punjab (Pakistan). Randomly selected 920 employees were provided with printed questionnaires. Data were gathered in three waves, with 6-month lags. The reason for collecting data in three waves was that minimum three waves are necessary to test true causal effects in a mediation model (Collins et al., 1998; Cole and Maxwell, 2003). In order to match responses of all three waves, each individual employee was assigned a distinct code. The study survey was initiated after having a written informed consent from the participants and approval from the Ethical Committee for Scientific Research at COMSATS, Vehari.

All waves of survey obtained employees’ self-ratings about relational coordination, job satisfaction, and the use of high-performance work practices. All study variables’ ratings were obtained in three waves. However, the data were used according to the need of analytical procedures. The control variables (gender, education, and tenure) were surveyed only in the first wave. After looking for missing values, 717 responses were usable from first-wave survey (78%).

In the second-wave survey, only those 717 employees were approached for whom we received usable responses in the first wave. However, two employees had left their jobs, and seven were on long-term leave. So, the second-wave questionnaires were distributed among 708 employees. After looking for missing values and matching the first- and second-wave responses, only 476 responses (67%) were usable. In Time 3, one out of 476 had left his job, and two were on long-term leave. Questionnaires were distributed to 473 employees. After looking for missing values and matching responses for the second and first wave, 322 (68%) usable responses were recorded. The response rate from initial sample to final usable data is 35%. Of the final 322 respondents, 185 were male (57%), and 137 (43%) were female. The mean age of respondents was 28 years, and the mean experience was 6 years. Respondent’s education was recorded as number of education years [≥18 years: 40 (12%); 16 years: 95 (30%); 14 years: 46 (14%); 12 years: 64 (20%); 10 years: 77 (24%)].

One may note a significant dropout of respondents from Wave 1 to Wave 3. Under such a situation, the issue of attrition bias may arise. However, attrition bias occurs “if participants who stay in a study differ from those who drop out” (Gustavson et al., 2012, p. 1). Following Brouer et al. (2011), we were able to ask branch managers to compare the characteristics of respondents and of those who did not respond in follow-up surveys. The managers informed that the respondents and non-respondents were alike in their characteristics (i.e., age, experience, education, gender). Moreover, in light of Miller and Wright (1995), we tested for attrition bias by applying independent-samples *t*-test to compare the characteristics of “those subjects who responded to all waves of the study with those who dropped out after only one wave” (p. 922). We performed an independent-samples *t*-test in the IBM (USA) Statistical Package for Social Sciences (SPSS) version 21. The results in Table 1 indicate that there was no significant difference in the characteristics of respondents and non-respondents with respect to gender, education, tenure, and age. So, attrition bias is less likely in our data.

**TABLE 1:** Independent *t*-test to compare the characteristics of respondents and non-respondents.

Group		*N*	Mean	Standard deviation	*t*-value	df	Significance (2-tailed)
Gender	Non-respondents	395	1.52	0.50	−1.55	715	0.12
	Respondents	322	1.57	0.50			
Education	Non-respondents	395	2.87	1.35	0.07	715	0.95
	Respondents	322	2.87	1.39			
Tenure	Non-respondents	395	5.87	1.31	−0.98	715	0.33
	Respondents	322	5.96	1.32			
Age	Non-respondents	395	28.11	7.01	0.29	715	0.77
	Respondents	322	27.96	6.97			

### Measures

Data were collected by using questionnaires already used in existing research. Relational coordination was measured by using employees’ self-ratings on Gittell et al. (2000) seven-item instrument where communication dimensions were scaled as follows: 1 = never, 2 = rarely, 3 = occasionally, 4 = often, 5 = always, and the relational dimensions were scaled as follows: for shared goals and mutual respect: 1 = not at all, 2 = a little, 3 = some, 4 = a lot, 5 = completely, and for shared knowledge; 1 = nothing, 2 = little, 3 = some, 4 = a lot, 5 = everything. High-performance work systems were measured by employees’ perceptions about their organization’s use of Gittell et al.’s (2010) six high-performance work practices: selection, conflict resolution, performance measurement, rewards, meetings, and use of boundary spanners. A five-point Likert scale was used (1 = never, 2 = rarely, 3 = occasionally, 4 = often, 5 = always) to measure employees’ self-ratings about using these practices. Peer justice climate was measured by using a five-item scale where four items were related to each of the justice dimensions, for example, distributive, procedural, interpersonal, and informational peer justice (Li et al., 2013; Molina et al., 2016), and one item was related to overall peer justice (Walumbwa et al., 2017). Job satisfaction was measured by using employees’ self-ratings on a four-item scale used in Eisenberger et al. (1997). For both peer justice and job satisfaction questionnaires, employees specified their level of agreement with each item on a five-point Likert scale (1 = strongly disagree, 5 = strongly agree).

Given that all study variables were measured by a single source (employees’ self-ratings), the issue of common method variance (CMV) may arise (Podsakoff et al., 2003). Common method variance is defined as the “variance that is attributable to the measurement method rather than to the constructs the measures represent” (Podsakoff et al., 2003, p. 879). While explaining “techniques for controlling common method biases,” Podsakoff et al. (2003) suggested that a potential remedy to control CMV “is to create a temporal separation by introducing a time lag between the measurement of the predictor and criterion variables” (p. 887). The issue of CMV is less likely in this study because we used time lags between the measurement of the predictor, mediator, and criterion variables. In addition, the variance inflation factor (VIF) generated in collinearity test also informs about the presence of CMV in data (Kock and Lynn, 2012; Kock, 2015). Moqbel and Kock (2018) suggested that VIF values lower than 3.3 indicate that CMV is not present in data. We performed a collinearity test before testing the hypothesized relationships in our model and found no VIF value higher than 3.3 (Table 4). So, it can be stated that CMV is not an issue in this study’s data.

### Control Variables

The effects of respondents’ gender (1, “male,” 2, “female”), age (in years), and tenure in the organization (in years) were controlled in direct and indirect effect models because these variables are likely to influence employee job satisfaction (Janssen and Van Yperen, 2004; Gittell et al., 2008).

### Analytical Approach

Data were analyzed by applying partial least squares structural equation modeling (PLS-SEM) in the latest version of SmartPLS software. Partial least squares path modeling, in relation to other SEM techniques such as covariance based or CB-SEM, is more desirable in social sciences as it is effective in analyzing small samples and non-normal data (Hair et al., 2014). As a component-based estimation technique, PLS-SEM uses iterative algorithms of least squares regressions (Hair et al., 2014; Haider et al., 2020). It is an appropriate technique for the studies that aim to make prediction and develop theories (Hair et al., 2016). Partial least squares SEM is preferred to other SEM techniques such as CB-SEM (covariance based SEM) because it can efficiently handle small samples and the data that are not normally distributed. Partial least squares SEM validates data in measurement model and tests the significance and relevance of hypothesized relationships in structural model.

## Results

### Evaluation of Measurement Model

Partial least squares measurement model has two types: reflective (principal factor) and formative (composite index) measurement models. The former type, where the “direction of causality is from construct to measure,” is considered appropriate for studies measuring perceptions, attitudes, and so on (Jarvis et al., 2003, p. 201). The evaluation of measurement model is performed by estimating internal consistency reliability, convergent validity (CV), and discriminant validity (DV) of survey instruments. Internal consistency reliability means that a construct’s all items are equally reliable. The values of Cronbach α (>0.70) and CR (between 0.70 and 0.90) are two standards for establishing IC in a latent construct. Table 2 shows that Cronbach α for all latent constructs is greater than 0.70. The CR values for all latent constructs are between 0.70 and 0.90, except for T1-HPWS and T1-JS. However, the values of these construct are less than 0.95, which is acceptable under a lenient criterion (Hair et al., 2014).

**TABLE 2:** Assessment of measurement model (construct reliability).

	Heterotrait–monotrait ratio												
	α	CR	AVE	1	2	3	4	5	6	7	8	9	10
1. Education	–	–	–										
2. Gender	–	–	–	0.10									
3. T1-PJC	0.76	0.84	0.51	0.18	0.13								
4. T1-HPWS	0.92	0.94	0.80	0.15	0.05	0.16							
5. T1-JS	0.74	0.82	0.54	0.29	0.03	0.15	0.08						
6. T1-RC	0.86	0.89	0.58	0.51	0.14	0.21	0.16	0.27					
7. T2-JS	0.86	0.91	0.71	0.27	0.09	0.17	0.07	0.21	0.27				
8. T2-RC	0.81	0.87	0.53	0.26	0.12	0.25	0.25	0.12	0.25	0.27			
9. T3-JS	0.86	0.90	0.70	0.35	0.10	0.30	0.26	0.24	0.32	0.63	0.55		
10. Tenure	–	–	–	0.22	0.07	0.15	0.06	0.11	0.09	0.05	0.08	0.08	

*α, Cronbach α; CR, composite reliability; AVE, average variance extracted; PJC, peer justice climate; HPWS, high-performance work systems; JS, job satisfaction; RC, relational coordination; T, time/wave.*

Convergent validity “is the extent to which a measure correlates positively with alternative measures of the same construct” (Hair et al., 2014, p. 102). Indicator reliability (factor loading of each indicator >0.70) and average variance extracted (AVE ≥ 0.50) are used for determining CV (Hair et al., 2014). The AVE values of all our latent constructs are greater than 0.50 (Table 2). The indicators T1-HPWS1, T1-HPWS6, T1-RC7, and T2-RC7 were deleted from their respective constructs because their factor loadings were less than 0.40 (Hair et al., 2014). Deletion, however, does not affect the meaning of a reflective construct (Jarvis et al., 2003). Factor loadings (λ) of some indicators (T1-PJC3, T1-JS1, T1-RC4, T2-R1, T2-RC5, and T2-RC 6) are less than 0.70 (Table 3). Nevertheless, these items were kept with their corresponding latent constructs as the “indicators with outer loadings between 0.40 and 0.70 should be considered for removal only if the deletion leads to an increase in composite reliability and AVE above the suggested threshold value” (Hair et al., 2014, p. 107). The deletion process was performed by using insights from Hair et al. (2014), and there was no increase in composite reliability and AVE above the suggested threshold value.

**TABLE 3:** Factor loadings.

PJC		T1-HPWS		T1-JS		T1-RC		T2-JS		T2-RC		T3-JS	

Ï	λ	Ï	λ	Ï	λ	Ï	λ	Ï	λ	Ï	λ	Ï	λ
T1-PJC1	0.74	T1-HPWS2	0.92	T1-JS1	**0.63**	T1-RC1	0.81	T2-JS1	0.82	T2-RC1	**0.60**	T3-JS1	0.74
T1-PJC2	0.76	T1-HPWS3	0.93	T1-JS2	0.73	T1-RC2	0.84	T2-JS2	0.86	T2-RC2	0.78	T3-JS2	0.83
T1-PJC3	**0.65**	T1-HPWS4	0.94	T1-JS3	0.77	T1-RC3	0.80	T2-JS3	0.86	T2-RC3	0.88	T3-JS3	0.92
T1-PJC4	0.70	T1-HPWS5	0.78	T1-JS4	0.80	T1-RC4	**0.56**	T2-JS4	0.82	T2-RC4	0.85	T3-JS4	0.86
T1-PJC5	0.70					T1-RC5	0.80			T2-RC5	**0.60**		
						T1-RC6	0.74			T2-RC6	**0.60**		

*Ï, indicators; λ, factor loadings; PJC, peer justice climate; HPWS, high-performance work systems; JS, job satisfaction; RC, relational coordination; T, time/wave.*

Discriminant validity is established to evaluate that the measures of one construct do not correlate with other constructs (Ringle et al., 2010). As a tradition, DV was established by using cross-loadings and Fornell and Larcker’s (1981) criterion. But these methods are insufficiently sensitive to detect DV. Henseler et al. (2015) introduced a more sensitive new criterion, heterotrait–monotrait ratio of correlations (HTMT), for measuring DV. We used this new criterion. Using a more conservative approach (considered as the strictest criterion), HTMT value between two constructs must be less than 0.85 (HTMT_0_._85_). Table 2 shows that all HTMT values between constructs are less than 0.85. So, DV has been established in our model.

### Evaluation of Structural Model

According to Hair et al. (2014), collinearity between each set of predictor variables must be checked before hypotheses testing. Partial least squares SEM also requires collinearity test at item level in formative measurement models. However, in case of reflective measurement model, collinearity test is not required at item level (see Hair et al., 2014). As we used reflective measurement model, the collinearity test was performed only at construct level. Variance inflation factor is a frequently used measure of collinearity. Its value should be 5 or lower. The SmartPls results in Table 4 indicate the absence of collinearity among the predictors because all VIF values are below 5.

**TABLE 4:** Collinearity assessment (inner VIF values).

	T2-JS	T2-RC	T3-JS
Education			1.19
Gender			1.03
T1-PJC		1.04	
T1-HPWS		1.04	1.07
T1-JS	1.05		
T1-RC	1.05	1.06	
T2-JS			1.11
T2-RC			1.13
T3-JS			
Tenure			1.08

*PJC, peer justice climate; HPWS, high-performance work systems; JS, job satisfaction; RC, relational coordination; T, time/wave.*

**FIGURE 2:**
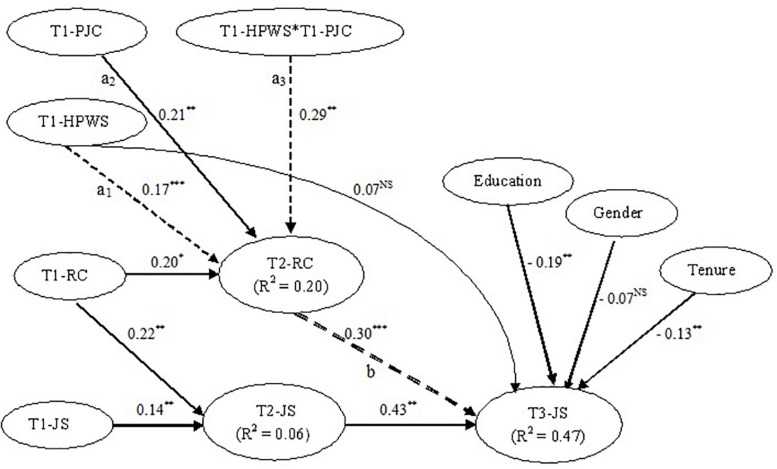
Estimated PLS longitudinal moderated-mediation path models. T1, Time 1; T2, Time 2; T3, Time 3; HPWS, high-performance work systems; JS, job satisfaction; RC, relational coordination; NS, non-significant. ^∗∗^*P* < 0.01; ^∗^*P* < 0.05.

Figure 2 shows the estimated longitudinal path model for mediation and moderated mediation where dotted lines indicate the hypothesized relationships after controlling for the respondents’ education, gender, and tenure and prior levels of study variables where applicable (solid lines). In this three-wave autoregressive model, the direct and indirect effects of independent variable on dependent variable take two-unit time lags (Maxwell et al., 2011). Two-unit time lag models allow to avoid half longitudinal designs (where one part of the model becomes cross-sectional; either the effect of predictor on mediator or the effect of mediator on outcome variable is measured at same point of time) (Cole and Maxwell, 2003; Haider et al., 2019b). In a three-wave longitudinal model, half longitudinal design is avoided by testing the effect of Time 1 predictor on Time 2 mediator and subsequent effect on Time 3 outcome variable (controlling for Time 1 mediator, and Time 1 and Time 2 outcome variable). Therefore, this study’s mediation hypothesis was examined by testing the effect of Time 1 HPWS (T1-HPWS) on Time 3 job satisfaction (T3-JS) through Time 2 relational coordination (T2-RC), controlling for the previous levels of relational coordination (T1-RC) and job satisfaction (T1-JS and T2-JS). Zhao et al.’s (2010) two-step process was followed to decide whether relational coordination mediated the HPWS–job satisfaction relationship.

To test moderated mediation, Time 1 peer justice climate (T1-PJC) and its interaction with Time 1 HPWS (T1-HPWS × T1-PJC) were added in the mediation model. Specifically, moderated mediation was examined by extending Hayes’ (2015) first stage moderation model to a three-wave longitudinal autoregressive mediation model. Based on Edwards and Lambert (2007) and Preacher et al. (2007), Hayes (2015) explained that first-stage model allows the effect of predictor on mediator in a mediation model to be moderated by another variable (moderator). This is the case in our research model.

### Mediation Results

As already mentioned, this study used a two-step mediation procedure developed by Zhao et al. (2010) and explained in Hair et al. (2016). The first step suggests that the indirect effect of predictor on dependent variable, via mediator, must be significant. Preacher et al.’s (2007) product of coefficients approach was used to estimate the coefficient of indirect effect. The significance of this effect was tested by using bias-corrected bootstrap confidence interval method for 5,000 samples. The results indicate (Figure 2) that the effect of HPWSs on job satisfaction, through relational coordination, is significant (β = 0.17^∗^0.30 = 0.05; *P* < 0.05).

The second step requires testing the direct effect of predictor on the dependent variable. Figure 2 shows that the direct effect of HPWS (T1-HPWS) on employee job satisfaction (T3-JS) is insignificant (β = 0.07; *P* > 0.05). Insights from existing literature (Cole and Maxwell, 2003; Rucker et al., 2011) suggest that the non-zero coefficient of direct effect indicates that relational coordination does not fully mediate the relationship between HPWSs and job satisfaction, and other possible mediators cannot be ignored. Given that the direct effect is non-zero and insignificant, and the indirect effect is significant, it can be stated that relational coordination mediates the relationship between HPWSs and job satisfaction (Zhao et al., 2010; Hair et al., 2016). It suggests that the mediator acquiesces well in our research model (supports Hypothesis 1).

### Moderated-Mediation Results

As discussed earlier, the test of moderated mediation was based on Hayes (2015) first-stage model where the effect of HPWSs on relational coordination (in our mediation model) was moderated by peer justice climate. As a tradition among researchers, the significance of moderating effect on predictor–mediator relationship is examined in a moderated-mediation model (Muller et al., 2005; Preacher et al., 2007; Hair et al., 2016). However, the latest literature on moderated mediation suggests that “a formal test of moderated mediation based on a quantification of the relationship between the proposed moderator and the size of the indirect effect is required to determine whether the indirect effect depends on the moderator” (Hayes, 2015, p.9). In other words, it is recommended to test the moderator’s effect on the indirect effect as a whole rather than testing an isolated moderating effect on independent variable–mediator relationship (Hayes, 2015).

In a first-stage autoregressive moderated-mediation model, the indirect effect of Time 1 predictor (T1-HPWS) on Time 3 outcome variable (job satisfaction: T3-JS) through Time 2 mediator (relational coordination: T2-RC) is the product of the conditional effect (i.e., T1-HPWS × T1-PJC) of Time 1 predictor (T1-HPWS) on Time 2 mediator (T2-RC), and the effect of Time 2 mediator (T2-RC) on Time 3 outcome variable (T3-JS). In other words, the product of path a_3_ and b in Figure 2 is the indirect effect in case of moderated mediation. This indirect effect (denoted as ω) can be written as follows:

(1) $$\omega =\left(a_{1}+a_{3}\text{PJC}\right)b$$

(2) $$\omega =a_{1}b+a_{3}b\text{PJC}$$

In the above equation, *a*_1_*b* is intercept, whereas *a*_3_*b* is slope. Based on Morgan-Lopez and MacKinnon (2006), Hayes (2015) calls *a*_3_*b* as “the index of moderated mediation,” which “is a quantification of the effect of (moderator) on the indirect effect of (predictor) on (outcome variable) through (mediator).” This index is the product of two path coefficients (*a*_3_ and *b* in Figure 2). Hayes (2015) states that a non-zero value of this index serves as a measure of moderated mediation and does not require “evidence of statistically significant interaction between any variable in the model and a putative moderator” (p. 3). In our model, the value of *a*_3_*b* (0.29 × 0.30 = 0.09) is non-zero, which indicates that the indirect effect of HPWSs on employee job satisfaction, through relational coordination, is not independent of peer justice climate but, rather, depends on it.

**FIGURE 3:**
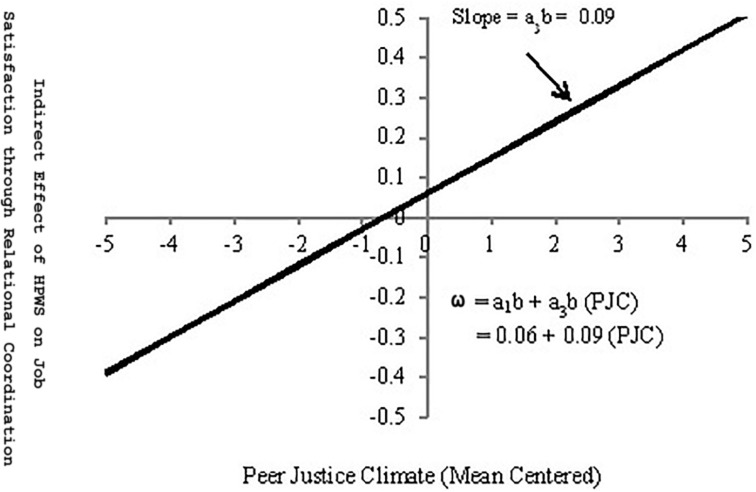
Graphical representation of Eq. 2 at different values of peer justice climate.

A non-zero index of moderated mediation means that the indirect effect is systematically larger or smaller for some values of (moderator) than others (Hayes, 2015, p. 4). By using some arbitrary values for moderator (peer justice climate), one can obtain a visual representation of the linear function (shown in Eq. 2). We used the arbitrary moderator values ranging from −5 to 5 (and the values of a_1_b and *a*_3_*b* from Figure 2) in Eq. 2 and obtained the linear function relating peer justice climate to the indirect effect of HPWSs on employee job satisfaction, through relational coordination (Figure 3). The positive slope of this function shows that the indirect effect of HPWSs on employee job satisfaction, through relational coordination, seems to increase with increase in employees’ perceptions of peer justice climate. The bootstrapping at 5,000 samples, in SmartPLS software, generated a biased corrected 95% bootstrap confidence interval (0.046–0.134) for the index of moderated mediation. This confidence interval has positive upper bound and does not include zero. Given that, it can be concluded that the indirect effect of HPWSs on employee job satisfaction, through relational coordination, is positively moderated by peer justice climate (supports Hypothesis 3).

## Discussion

The aim of this study was to provide an explanation for why HPWSs affect employee job satisfaction. We examined whether HPWSs influence job satisfaction by affecting the degree to which employees exhibit relational coordination and whether this coordination prompts job satisfaction. Consistent with existing research, the findings of this study provide support for the direct relationship between HPWSs and job satisfaction (Messersmith et al., 2011; García-Chas et al., 2016; Liu et al., 2016; Ogbonnaya and Valizade, 2018).

However, previous research on HPWS–job satisfaction relationship is dominated by the studies using cross-sectional design and informs little about the temporal effects of HPWSs on job satisfaction. Contrary to previous research, this study draws more accurate causal inferences by controlling for prior levels of job satisfaction and HPWSs in a longitudinal design. Given that, this study addresses the issue of scarce longitudinal research on the association between HPWSs and employee job satisfaction.

The results also provide support for the relationship between HPWSs and relational coordination. This finding is consistent with Gittell et al. (2010) and Riaz (2016), who found that high-performance work practices enhance relational coordination among employees. The longitudinal results also support the relationship between relational coordination and employee job satisfaction. This result is consistent with Gittell et al. (2008) and Margalina et al. (2014). Overall, the research on empirical examination of the aforementioned relationships is scarce. Moreover, previous research has used cross-sectional rather than longitudinal or experimental design and remains unable to draw true causal inferences. This study has addressed this issue.

An important aspect of this research is that it answered the question: Do HPWSs enhance relational coordination, and does increase in relational coordination lead to employee job satisfaction? By incorporating the mediating variable, we observed a strong support for a partially mediated model of the relationship between HPWSs and job satisfaction. Our research concludes that HPWSs themselves are less explicative in describing their effect on employee job satisfaction. Other mechanisms such as relational coordination can explain why HPWSs explain job satisfaction.

Previous research examined the mediating mechanisms between HPWS–job satisfaction relationship in cross-sectional study designs, which are not well suited to test mechanisms, that is, sets of causal effects (Cole and Maxwell, 2003; Maxwell and Cole, 2007). The use of longitudinal design has enabled us to determine true causal relationships in the mediation process. To our knowledge, this is the first longitudinal study examining the mediating effect of relational coordination in the relationship between HPWSs and employee job satisfaction.

The results of mediating hypothesis (Hypothesis 2) are in line with the Gittell et al.’s (2010) model of HPWSs and other theoretical insights provided while developing argument for this hypothesis. Empirical results confirm that HPWSs increase relational coordination among coworkers, and consequently, this increased relational coordination enhances their job satisfaction. By focusing on the mediating relationship, this study expands Gittell et al.’s (2008) and Gittell et al.’s (2010) work by confirming that HPWSs enhance relational coordination, and relational coordination enhances employee job satisfaction. However, our research is first in examining the effect of relational coordination as a mediating mechanism between HPWSs and job satisfaction.

Moreover, the results of Hypothesis 3 explain that the indirect effect of HPWSs on employee job satisfaction through relational coordination is moderated by peer justice climate. It suggests that the way coworkers treat each other affects how well they communicate and develop relationships in a work setting. Subsequently, it affects their job satisfaction. In other words, peer justice climate enhances fit between HPWSs and relational coordination as it provides a positive contingency in organizations. Although previous research has examined the moderating effect of justice climate on diverse relationships in organizations (Li et al., 2013), no previous study examined the moderating effect of peer justice climate on indirect effects. This study’s examination of moderating effect on indirect relationship is important because the mediating mechanism such as relational coordination is likely to exert greater effect of employee job satisfaction as it receives greater effect of organizational policies (such as HPWSs) in the presence of peer justice climate.

There are some interesting findings about the effect of control variables on employee job satisfaction. We controlled for the level of education, gender, and tenure of the respondents and found negative effect of all these variables on job satisfaction. In existing literature, the relationship between education and job satisfaction is mix and inconclusive (González et al., 2016). In our research, the negative relationship between education and job satisfaction is consistent with existing research such as Clark et al. (1996); Gazioglu and Tansel (2006), Grund and Slivka (2001), Sloane and Williams (1996), Allen and Van der Velden (2001), while inconsistent with the findings of other research, which found a positive relationship between education and job satisfaction (Lydon and Chevalier, 2002; Nikolaou et al., 2005). As suggested by González et al. (2016), the negative effect in our study may be due to high expectations of highly qualified employees. We also found a negative (but insignificant) relationship between gender and job satisfaction. Consistent with existing research, this may be due to the fact that for an identical job men’s expectation tends to be higher than women’s (Clark, 1997). Regarding the effect of age on job satisfaction, previous research shows an increase in job satisfaction as age increases (Saleh and Otis, 1964). A recent study by Taiwo et al. (2017) shows that the level of job satisfaction decreases as respondents’ age moves from 25 to 30 years. So, the negative relationship in our model is consistent with existing research because average age of our respondents is 28 years, which reflects a relatively young study sample.

### Theoretical Implications

We believe that this study contributes to organizational behavior and human resource management literature in four ways. First, after a scholarly discussion on the effect of HPWSs on the dimensions of relational coordination and subsequent effect of these dimensions on employee job satisfaction, we provided theoretical reasoning as to why relational coordination mediates the relationship between HPWSs and job satisfaction. It is important because no previous research has developed such argument. Empirical examination of this mediating effect unfolds how HPWSs exert their effect of employee job satisfaction. In strategic HRM literature, one of the highly debated issues is to understand the mediating processes that explain why HPWSs affect employee and organizational outcomes (Buller and McEvoy, 2012; Agarwal and Farndale, 2017). Our analysis of mediating mechanism is a strong contribution in this debate. High-performance work systems are effective in enhancing employee job satisfaction because they provide employees with relational resources. This, in fact, draws researchers’ attention toward the synergic effect of HPWSs on several mediators that, successively, affect employee and organizational outcomes (Delery and Doty, 1996; Agarwal and Farndale, 2017; Beltrán-Martín et al., 2017). Second, this study extends literature on the consequences of HPWSs by examining the boundary effect of peer justice climate on the aforementioned indirect relationship. Third, by testing the moderating effect of peer justice climate, this study has added in the scarce literature on the antecedents of relational coordination. Finally, this study has examined true causal relationships in a moderated-mediation model by using longitudinal rather than cross-sectional design.

### Practical Implications

It is well recognized that human capital is a valuable source of competitive advantage. Besides human resource development, organizations need to retain these resources (Riaz, 2016). Satisfied employees are more likely to stay with the organization and perform better as they have greater organizational commitment (Whitener, 2001; Allen et al., 2003). This study’s results suggest that organizations can achieve employee job satisfaction by implementing those practices that help foster relational coordination among coworkers. More specifically, managers’ use of conflict resolution practice may hinder the situation of disrespect among coworkers. It is important because an environment of mutual respect promotes positive interactions among employees and, consequently, the job satisfaction (Trivellas et al., 2015). Overall, managers’ use of meetings, rewards, and performance measurement can be effective in increasing communication, knowledge, and goal sharing among employees. Furthermore, the findings of this study suggest that managers should develop a climate of peer justice for achieving greater benefits from the application of high-performance work practices. In this regard, managers can help employees to improve peer justice climate by promoting dignity and respect and reducing biases among coworkers.

### Limitations and Future Research

Despite its theoretical contribution and practical implications, our research does have some limitations. First, the issue of CMV may arise as the data were collected from a single source (Podsakoff et al., 2003). However, this bias can be controlled if longitudinal survey design is used (Malhotra et al., 2017). This study not only used a longitudinal design but also estimated the VIF to detect CMV. So, it is less likely that common method bias might have affected the results of this study. Future researchers can strengthen their findings by collecting data from multiple sources. Second, our sample was from the bank branches of Vehari district (Pakistan), and this context may be idiosyncratic enough to restrict the external viability of our results. Future studies can extend the findings of this research in other sectors and regions.

While theory and evidence support our research model, we cannot ignore other possible illustrations of our results. For instance, satisfied employees may be more likely to exhibit relational coordination and, consequently, may attain greater attention from management to participate in meetings and conflict resolution activities and receive rewards and positive performance appraisal. Because social exchanges are basic to relational coordination, we recognize that the process may be reciprocal. One recommendation for future scholars is to establish and examine a more comprehensive characterization of the viable antecedents of relational coordination and to also discover the level of mutual cause–effect relationship.

Despite these limitations, we surmise that we have reached the objective of this study. First, we provided an explication for why HPWSs may affect employee job satisfaction. The obvious process is that HPWSs influence the degree to which employees exhibit relational coordination, and this coordination prompts job satisfaction. Second, this study has also developed and supported the role of HPWSs and peer justice climate as antecedents of relational coordination. Although we did not test other predictors of relational coordination, it can be suggested that efforts to advance the use of HPWSs may be effective in conveying how an organization values and promotes employee relationships and job satisfaction.

## Conclusion

This study concludes that HPWSs themselves are less explicative in describing their effect on employee job satisfaction. Other mechanisms such as relational coordination and peer justice climate can explain why and when HPWSs explain job satisfaction.

## Data Availability Statement

The datasets used for this study are available on request to the corresponding author.

## Ethics Statement

The study survey was initiated after having a written informed consent from the participants, and approval from the Ethical Committee for Scientific Research (ECSR) at COMSATS, Vehari.

## Author Contributions

SH initiated the basic idea and wrote the main part of the manuscript. CD-P-H built the article structure. MD-P-H improved the manuscript.

## Conflict of Interest

The authors declare that the research was conducted in the absence of any commercial or financial relationships that could be construed as a potential conflict of interest.
